# A polygenic risk score for multiple myeloma risk prediction

**DOI:** 10.1038/s41431-021-00986-8

**Published:** 2021-11-30

**Authors:** Federico Canzian, Chiara Piredda, Angelica Macauda, Daria Zawirska, Niels Frost Andersen, Arnon Nagler, Jan Maciej Zaucha, Grzegorz Mazur, Charles Dumontet, Marzena Wątek, Krzysztof Jamroziak, Juan Sainz, Judit Várkonyi, Aleksandra Butrym, Katia Beider, Niels Abildgaard, Fabienne Lesueur, Marek Dudziński, Annette Juul Vangsted, Matteo Pelosini, Edyta Subocz, Mario Petrini, Gabriele Buda, Małgorzata Raźny, Federica Gemignani, Herlander Marques, Enrico Orciuolo, Katalin Kadar, Artur Jurczyszyn, Agnieszka Druzd-Sitek, Ulla Vogel, Vibeke Andersen, Rui Manuel Reis, Anna Suska, Hervé Avet-Loiseau, Marcin Kruszewski, Waldemar Tomczak, Marcin Rymko, Stephane Minvielle, Daniele Campa

**Affiliations:** 1 Genomic Epidemiology Group, German Cancer Research Center (DKFZ), Heidelberg, Germany; 2 Department of Biology, University of Pisa, Pisa, Italy; 3 Department of Hematology, University Hospital of Cracow, Cracow, Poland; 4 Department of Hematology, Aarhus University Hospital, Aarhus, Denmark; 5 Hematology Division, Chaim Sheba Medical Center, Tel Hashomer, Israel; 6 Department of Hematology, Sea Hospital, Gdynia, Poland; 7 Department of Internal and Occupational Diseases, Hypertension and Clinical Oncology, Medical University Wroclaw, Wroclaw, Poland; 8 Cancer Research Center of Lyon/Hospices Civils de Lyon, Lyon, France; 9 Hematology Clinic, Holycross Cancer Center, Kielce, Poland; 10 Department of Hematology, Institute of Hematology and Transfusion Medicine, Warsaw, Poland; 11 Genomic Oncology Area, GENYO. Centre for Genomics and Oncological Research: Pfizer, University of Granada/Andalusian Regional Government, Granada, Spain; 12 Hematology department, Virgen de las Nieves University Hospital, Granada, Spain; 13 Third Department of Internal Medicine, Semmelweis University, Budapest, Hungary; 14 Department of Internal and Occupational Diseases, Medical University Wroclaw, Wroclaw, Poland; 15 Department of Hematology, Odense University Hospital, Odense, Denmark; 16 Institut Curie, PSL Research University, Mines ParisTech Inserm, U900 Paris, France; 17 Hematology Department, Teaching Hospital No 1, Rzeszów, Poland; 18 Department of Hematology, Rigshospitalet, Copenhagen University, Copenhagen, Denmark; 19 Clinical and Experimental Medicine, Section of Hematology, University of Pisa, Pisa, Italy; 20 Department of Haematology, Military Institute of Medicine, Warsaw, Poland; 21 Department of Hematology, Rydygier Specialistic Hospital, Cracow, Poland; 22 Life and Health Sciences Research Institute (ICVS), School of Health Sciences/Molecular Oncology Research Center, University of Minho, Braga, Portugal; 23 Department of Hematology, Jagiellonian University Medical College, Cracow, Poland; 24 Maria Sklodowska-Curie National Research Institute of Oncology, Warsaw, Poland; 25 National Research Centre for the Working Environment, DK-2100 Copenhagen, Denmark; 26 Institute of Molecular Medicine, University of Southern Denmark, Odense, Denmark; 27 ICVS/3B’s - PT Government Associate Laboratory, Braga/Guimarães, Portugal; 28 Molecular Oncology Research Center, Barretos Cancer Hospital, S.Paulo, Brazil; 29 Unité de Génomique du Myélome, Institut Universitaire du Cancer Toulouse – Oncopole, Toulouse, France; 30 Department of Hematology, University Hospital Bydgoszcz, Bydgoszcz, Poland; 31 Medical University of Lublin, Lublin, Poland; 32 Department of Hematology, N. Copernicus Town Hospital, Torun, Poland; 33 CRCINA, INSERM, CNRS, Université d’Angers, Université de Nantes, Nantes, France

**Keywords:** Cancer genomics, Cancer epidemiology

## Abstract

There is overwhelming epidemiologic evidence that the risk of multiple myeloma (MM) has a solid genetic background. Genome-wide association studies (GWAS) have identified 23 risk loci that contribute to the genetic susceptibility of MM, but have low individual penetrance. Combining the SNPs in a polygenic risk score (PRS) is a possible approach to improve their usefulness. Using 2361 MM cases and 1415 controls from the International Multiple Myeloma rESEarch (IMMEnSE) consortium, we computed a weighted and an unweighted PRS. We observed associations with MM risk with OR = 3.44, 95% CI 2.53–4.69, *p* = 3.55 × 10^−15^ for the highest vs. lowest quintile of the weighted score, and OR = 3.18, 95% CI 2.1 = 34–4.33, *p* = 1.62 × 10^−13^ for the highest vs. lowest quintile of the unweighted score. We found a convincing association of a PRS generated with 23 SNPs and risk of MM. Our work provides additional validation of previously discovered MM risk variants and of their combination into a PRS, which is a first step towards the use of genetics for risk stratification in the general population.

## Introduction

Multiple myeloma (MM) is the third most common hematological malignancy with a worldwide incidence rate of 2.1/100,000 new cases each year (https://gco.iarc.fr/today/home) [1]. MM is preceded by monoclonal gammopathy of undetermined significance (MGUS), an asymptomatic premalignant condition [2, 3], and by smoldering myeloma (SM), a more advanced precursor of the disease [4].

MM etiology has a strong genetic component, with several variants associated with its risk [5–21]. In particular, genome-wide associations studies (GWAS) identified 23 MM risk loci, but as for many other traits the individual penetrance of each SNP is low, with odds ratios (OR) per risk allele ranging from 1.11 to 1.38 [5, 7, 14, 15, 17].

Considering also the rarity of the disease, the identified variants have a poor clinical use in predicting the individual risk, especially if considering the general population. A possible approach to improve usefulness of genetic risk markers could be to combine the SNPs in a polygenic risk score (PRS) in order to have a better estimation of their cumulative effect on the risk of developing the disease. This method has been successfully applied to several diseases including breast, prostate, colorectal, and pancreatic cancer [22–28]. For myeloma, a PRS was briefly mentioned in the latest GWAS publication [17]. An earlier study compared a 16-SNP PRS in familial and sporadic MM cases [29]. A PRS including all the known risk SNPs has been also evaluated in African–Americans [30].

The aim of this work is to use the International Multiple Myeloma (IMMeNSE) consortium to establish a PRS for MM and provide an evaluation of the PRS performance in an independent set of MM cases and controls.

## Materials and methods

### Study population

We used DNA samples from 2361 MM patients and 1415 controls from 7 countries (Denmark, France, Hungary, Israel, Italy, Poland, and Portugal) within the IMMEnSE consortium [6], for whom information on sex and age was available. Cases were defined by a confirmed diagnosis of MM according to the International Myeloma Working Group criteria [31]. Controls were selected from the general population, from hospitalized subjects with different diagnoses excluding cancer, or from blood donors. Characteristics of the study population are summarized in Table 1.

**Table 1:** Description of the study population.

	Cases	Controls	Total
Country			
Denmark	299	478	777
France	467	176	643
Hungary	104	81	185
Israel	81	68	149
Italy	251	224	475
Poland	1034	267	1301
Portugal	125	121	246
Total	2361	1415	3776
Sex			
Male	52.6%	52.4%	52.5%
Female	47.4%	47.6%	47.5%
Median age	61	50	58

### SNP selection

To build the PRS we used 23 SNPs shown to be associated with MM risk at genome-wide significance level (*p* < 5 × 10^−8^) by previous GWAS [5, 7, 14, 15, 17]. We did not include variants reported to be associated with MM risk but not at genome-wide level of significance (e.g., those reported by Erickson et al. [9]). Characteristics of the SNPs included in the PRS are summarized in Supplementary Table 1.

### Genotyping and PRS computation

Genotyping was performed using TaqMan technology (ThermoFisher Applied Biosystems, Waltham MA, USA) according to the manufacturer’s recommendations. TaqMan assays were not available for some SNPs, therefore we replaced them with surrogates in high linkage disequilibrium (*r*^2^ > 0.9), as detailed in Supplementary Table 1.

For each SNP, the number of alleles associated with higher MM risk were counted and added up for each study subject, resulting in an unweighted PRS, which had a theoretical range from 0 (no MM risk alleles) to 46 (all risk alleles are present at each SNP in homozygosity). In addition, we built a weighted PRS by using the ORs of the codominant model of the association of each variant with MM risk in the IMMEnSE population as coefficients to weight the relative effects of the risk SNPs. For each SNP in the weighted PRS, a value of 0 was assigned if 0 risk alleles were present, the ln(OR) of the heterozygous was assigned if one risk allele was present, and the ln(OR) of the homozygous was assigned if two risk alleles were present. Then all the values were summed among them for each subject. We built alternative weighted PRSs by using ORs from the literature, or values calculated in our dataset. Only a subset of the study subjects (1426 cases and 969 controls) had a 100% SNP call rate. Therefore, in order to be able to compute comparable score values for all study subjects, we also considered “scaled” scores, in which the PRS values for each subject were multiplied by the ratio between 23 (total number of SNPs) and the number of effectively genotyped SNPs for the subject in question. For both PRSs (weighted and unweighted), we calculated quintiles based on the distribution of values in the controls.

The formulas for the unweighted and weighted scores are respectively $\mathop {\sum}\nolimits_1^m {aj}$ and $\mathop {\sum}\nolimits_1^m {aXj}$, where *a* = number of risk alleles (0, 1, 2), *m* = total number of SNPs (23), *j* = *j*th subject, *X* = ln(OR). Supplementary Table 2 shows an example of how the scores were generated.

### Data filtering and statistical analysis

Samples with call rate less than 80% were not included in subsequent analysis. Pearson chi square was used to test departure from Hardy–Weinberg equilibrium (HWE) in the overall control group and in the individual countries.

To validate the associations between the individual SNPs and MM risk, we used logistic regression according to the log-additive and codominant models, using the more common allele in controls as the reference category.

We analyzed the association between the PRSs and MM risk by logistic regression. Age-stratified analyses were performed by comparing all controls with younger or older cases, with cutpoints at 55 (to distinguish between early onset and non-early onset cases), 61 (median age at onset of the cases in this study), or 69 years of age (median age at onset of MM, https://seer.cancer.gov/statfacts/html/mulmy.html) [32]. All analyses were adjusted for age, sex, and geographic region of origin.

We set up receiver operating characteristic (ROC) curves and calculated the areas under the curve (AUC), to determine the performance of the PRSs in discriminating MM cases from individuals without the disease.

## Results

We genotyped a total of 3376 subjects (2361 cases and 1415 controls). Controls from Portugal resulted out of HWE for SNPs rs877529 and rs4325816 in one 384-well plate (using a Bonferroni-corrected threshold of *p* < 0.002). Therefore, genotypes of Portuguese subjects for those two SNPs were dropped from the dataset. The remaining data were used for further statistical analyses. Duplicated samples (8% of the total) showed a concordance rate higher than 99%.

The associations between 12 of the SNPs and MM risk were replicated in IMMEnSE (*p* < 0.05) (Table 2). Regardless of statistical significance, all SNPs showed ORs going in the same directions as originally reported in the literature.

**Table 2:** Association between the selected SNPs and MM risk in the IMMEnSE population.

SNP	Cases			Controls			Log-additive model			Codominant model						
							M vs. m			MM vs. Mm			MM vs. mm			*p* trend
	MM	Mm	mm	MM	Mm	mm	OR	95% CI	*p*	OR	95% CI	*p*	OR	95% CI	*p*	
rs6746082	1542	588	93	896	422	68	0.89	0.78–1.02	0.098	0.90	0.76–1.07	0.260	0.76	0.52–1.12	0.165	0.481
rs4325816	1423	690	91	818	431	79	0.89	0.78–1.02	0.092	0.95	0.80–1.12	0.529	**0.69**	**0.48**–**0.99**	**0.048**	0.197
rs1052501	1351	817	130	924	406	46	**1.28**	**1.17**–**1.55**	**3.1** × **10**^−**5**^	**1.43**	**1.21**–**1.70**	**3.2** × **10**^−**5**^	1.50	1.00–2.24	0.050	**1.1** × **10**^−**4**^
rs10936599	1435	769	115	796	501	88	**0.84**	**0.73**–**0.95**	**0.007**	**0.83**	**0.71**–**0.99**	**0.034**	**0.70**	**0.49**–**0.99**	**0.044**	0.121
rs2548594	1326	808	157	773	511	99	0.94	0.82–1.06	0.298	0.97	0.82–1.14	0.679	0.83	0.60–1.13	0.237	0.213
rs6595443	682	1119	465	497	619	266	**1.15**	**1.03**–**1.29**	**0.011**	**1.29**	**1.08**–**1.55**	**0.004**	**1.29**	**1.03**–**1.60**	**0.027**	**0.017**
rs34229995	2136	134	10	1339	63	2	1.32	0.95–1.83	0.102	1.25	0.87–1.80	0.230	2.71	0.54–13.54	0.225	0.434
rs2285803	975	981	292	666	598	134	**1.15**	**1.02**–**1.29**	**0.022**	1.08	0.91–1.28	0.365	**1.40**	**1.07**–**1.83**	**0.013**	**0.007**
rs9373839	1492	734	93	881	388	49	1.03	0.90–1.19	0.639	1.09	0.92–1.30	0.322	0.91	0.60–1.37	0.640	0.767
rs4487645	1280	871	152	614	625	158	**0.72**	**0.64**–**0.82**	**1.7** × **10**^−**7**^	**0.74**	**0.63**–**0.87**	**3.6** × **10**^−**5**^	**0.51**	**0.38**–**0.68**	**3.3** × **10**^−**6**^	**2.4** × **10**^−**6**^
rs17507636	1315	813	144	715	540	136	**0.78**	**0.69**–**088**	**6.2** × **10**^−**5**^	**0.79**	**0.67**–**0.93**	**0.005**	**0.59**	**0.44**–**0.80**	**0.001**	**0.001**
rs2170352	1254	847	182	770	538	85	1.08	0.96–1.23	0.210	1.02	0.86–1.20	0.839	1.31	0.96–1.82	0.091	0.431
rs7781265	1816	474	40	1075	269	18	1.08	0.91–1.28	0.383	1.05	0.87–1.28	0.601	1.35	0.70–2.59	0.373	0.637
rs1948915	973	1048	287	639	611	150	**1.60**	**1.03**–**1.30**	**0.012**	1.13	0.96–1.33	0.151	**1.38**	**0.96**–**1.33**	**0.014**	**0.011**
rs2811710	1085	951	261	548	578	199	**0.83**	**0.74**–**0.94**	**0.002**	0.86	0.73–1.02	0.092	**0.68**	**0.53**–**0.87**	**0.002**	0.117
rs7187359	1108	905	248	733	534	129	1.08	0.96–1.21	0.204	1.05	0.89–1.24	0.593	1.21	0.92–1.59	0.175	0.137
rs2790454	1310	836	157	768	527	103	0.92	0.81–1.04	0.178	0.95	0.80–1.12	0.522	0.80	0.58–1.09	0.152	0.201
rs7193541	884	1069	367	480	644	256	0.93	0.83–1.03	0.177	0.87	0.73–1.04	0.124	0.88	0.70–1.10	0.271	0.722
rs4273077	1626	554	53	1042	260	24	**1.26**	**1.07**–**1.49**	**0.006**	**1.29**	**1.06**–**1.57**	**0.010**	1.41	0.81–2.48	0.227	0.371
rs11086029	1336	754	107	854	450	62	1.14	1.00–1.31	0.052	1.15	0.97–1.36	0.116	1.31	0.89–1.91	0.167	0.217
rs6066835	1924	374	44	1159	229	7	1.15	0.95–1.39	0.162	1.07	0.87–1.32	0.500	**2.86**	**1.05**–**7.80**	**0.040**	0.884
rs138745	970	1058	296	597	619	172	1.10	0.98–1.23	0.107	1.08	0.91–1.28	0.372	1.23	0.95–1.57	0.111	0.397
rs877529	678	986	486	471	565	254	**1.22**	**1.09**–**1.37**	**3.8** × **10**^−**4**^	1.21	1.01–1.46	0.440	**1.50**	**1.20**–**1.88**	**4.1** × **10**^−**4**^	**1.4** × **10**^−**4**^

All analyses were adjusted for age, sex, and geographic region of origin. Results in bold are statistically significant (*p*  < 0.05).

*M* major allele, *m* minor allele, *OR*  odds ratio, *CI* confidence interval, as calculated in IMMEnSE.

We observed strong associations between the PRS and MM risk (Table 3). When we computed the association between the PRSs and MM risk considering only 1426 cases and 969 controls with a call rate of 100%, we observed an OR = 3.18, 95% CI 2.34–4.33, *p* = 1.62 × 10^−13^ for the highest vs. lowest quintile of the unweighted score and OR = 3.44, 95% CI 2.53–4.69, *p* = 4.86 × 10^−15^ for the highest vs. lowest quintile of the weighted score. Results were very similar when we considered the whole dataset including 2361 cases and 1415 controls and “scaled” PRSs (Table 3), as well as when we built weighted scores using ORs for each SNP from the original GWASs (Table 3).

**Table 3:** Associations between PRSs and MM risk with the different types of scores.

Type of score	Quintiles	OR^a^	95% CI^a^	*p*_value_
Unweighted, subjects with 100% call rate	1	1.00	–	Ref.
	2	0.63	0.46–0.86	0.004
	3	3.16	2.31–4.31	4.33 × 10^−13^
	4	2.42	1.81–3.24	3.17 × 10^−9^
	5	3.18	2.34–4.33	1.62 × 10^−13^
	Continuous^b^	1.43	1.34–1.54	7.00 × 10^−23^
Unweighted scaled, all subjects	1	1.00	–	Ref.
	2	1.52	1.17–1.97	0.002
	3	1.44	1.13–1.83	0.003
	4	2.20	1.73–2.80	1.45 × 10^−10^
	5	2.93	2.28–3.78	9.00 × 10^−16^
	Continuous^b^	1.29	1.22–1.37	1.00 × 10^−17^
Weighted, subjects with 100% call rate^c^	1	1.00	–	Ref.
	2	1.33	0.95–1.86	0.096
	3	1.60	1.15–2.23	0.005
	4	2.43	1.77–3.35	4.78 × 10^−8^
	5	3.44	2.53–4.69	3.55 × 10^−15^
	Continuous^b^	1.37	1.28–1.46	2.00 × 10^−18^
Weighted scaled, all subjects^c^	1	1.00	–	Ref.
	2	1.29	0.98–1.70	0.068
	3	1.53	1.17–2.01	0.002
	4	2.24	1.72–2.91	1.68 × 10^−9^
	5	3.12	2.42–4.02	2.00 × 10^−17^
	Continuous^b^	1.33	1.26–1.41	3.00 × 10^−22^
Weighted 100% call rate using GWAS OR^d^	1	1.00	–	Ref.
	2	1.18	0.84–1.65	0.334
	3	1.56	1.12–2.17	0.008
	4	2.17	1.59–2.97	1.29 × 10^−6^
	5	3.24	2.39–4.39	3.93 × 10^−14^
	Continuous^b^	1.35	1.27–1.45	2.00 × 10^−17^
Weighted scaled using GWAS OR^d^	1	1.00	–	Ref.
	2	1.21	0.93–1.60	0.161
	3	1.56	1.20–2.04	0.001
	4	2.02	1.57–2.62	7.86 × 10^−8^
	5	2.89	2.25–3.71	9.00 × 10^−16^
	Continuous^b^	1.31	1.24–1.38	9.00 × 10^−20^

^a^*OR* odds ratio; *CI* confidence interval; all analyses were adjusted for age, sex and geographic region of origin.

^b^The unit for the analysis with the continuous variable was the increment of one quintile.

^c^The weights used to build this score were the ORs of the associations between the individual SNPs and MM risk observed in the IMMEnSE population.

^d^The weights used to build this score were the ORs of the associations between the individual SNPs and MM risk observed in the literature.

A histogram showing the difference in number of risk alleles (unweighted PRS) between cases and controls is shown in Supplementary Fig. 1.

In order to focus on the extreme parts of the risk distribution, we also calculated the difference in risk of subjects in the 95th percentile compared to subjects in the 5th percentile, and we found a substantial difference in risk (OR = 5.77, 95% CI 2.37–14.06, *p* = 1.12 × 10^−4^). Furthermore, we compared the subjects in the 95th percentile with subjects in the middle of the score distribution (third quintile) and we obtained an OR = 4.22, 95% CI 2.11–8.44, *p* = 4.52 × 10^−5^. All the tail distribution results are shown in Table 4.

**Table 4:** Associations between subjects in the 95^th^ percentile vs 5^th^ and third quintile and MM risk with the different types of scores.

Type of score	No of cases	No of controls	Distribution	OR^a^	95% CI^a^	*p*_value_
Unweighted 100% call rate	202	44	95% vs 5%	5.77	2.37–14.06	1.12 × 10^−4^
	476	142	95% vs third quintile	4.22	2.11–8.44	4.52 × 10^−5^
Unweighted scaled	356	141	95% vs 5%	4.12	2.42–7.01	1.81 × 10^−7^
	745	407	95% vs third quintile	3.05	2.15–4.32	3.73 × 10^−10^
Weighted 100% call rate	221	97	95% vs 5%	6.81	3.52–13.16	1.20 × 10^−8^
	398	241	95% vs third quintile	3.05	1.98–4.70	4.41 × 10^−7^
Weighted scaled	316	141	95% vs 5%	4.29	2.52–7.30	7.95 × 10^−8^
	646	352	95% vs third quintile	2.41	1.68–3.45	1.64 × 10^−6^

In addition, we performed case-control analyses stratifying the cases by age at diagnosis. We used three age cutpoints: 55, 61, and 69. The PRS was associated with MM risk in all strata, without differences in risk due to age of onset (data not shown).

The AUCs for each score are shown in Table 5. The best performance was observed for the unweighted PRS when considering only subjects with 100% call rate (AUC = 0.64, 95% CI = 0.62–0.67).

**Table 5:** Areas under the curve (AUC) for each PRS.

	AUC	95% CI
Unweighted score		
Subjects with call rate = 100%	0.644	0.622–0.666
“Scaled” score, all subjects	0.601	0.583–0.619
Weighted score calculated using ORs estimated in IMMEnSE		
Subjects with call rate = 100%	0.628	0.605–0.650
“Scaled” score, all subjects	0.615	0.597–0.633
Weighted score calculated using ORs from published GWAS		
Subjects with call rate = 100%	0.628	0.606–0.650
“Scaled” score, all subjects	0.609	0.591–0.627

## Discussion

Twenty-three SNPs affecting risk of MM were identified through GWAS. Since individually they do not explain a large proportion of the disease risk, we combined them in a PRS, which showed association with MM risk with strong statistical significance. Our results are encouraging, since when comparing the tails of the PRS distribution we observed a fourfold or more increase in risk.

The best area under the curve associated with the PRS was modest (AUC = 0.64, 95% CI = 0.62–0.67). However, this test could show a much better predictive ability in a selected population at already increased risk, such as individuals with MGUS or SM patients. We expect that the PRS performance will improve as more variants associated with MM are discovered, as shown by studies on other cancer types [23, 26, 27]. A further step to the clinical use of PRS is to combine them with environmental or lifestyle risk factors, as well as family history. We can envisage that in the middle/long term an enhanced MM risk PRS could become a powerful prediction tool for individualized risk stratification. Genotyping of risk loci will be done quickly and inexpensively in large groups of the population. Information on risk loci will be combined with questionnaire data on non-genetic risk factors, and specialized algorithms will estimate disease risk in a personalized manner. This will allow to adopt preventive measures, such as enhanced surveillance or intensified screening of people at high risk.

A limitation of this work is that the individuals used are all of European origin, making it difficult to generalize the data for other ethnicities. The same PRS was recently studied in African–Americans, with results comparable to those of European descent people [30]. Another limitation is that we examined only genetic polymophisms. It would be worth exploring whether a multifactorial score including also non-genetic risk factors could have a better predictive power. Unfortunately, we do not have complete data about known MM risk factors in IMMEnSE, therefore we can not explore multifactorial risk scores with meaningful numbers of cases and controls.

In conclusion, we found a convincing association of a 23-SNP PRS and MM risk. Our work provides additional validation of previously discovered MM risk variants and of their combination into a PRS, which is a first step toward the use of genetic background in the prevention of the disease. Additional risk SNP discovery will allow to generate PRS with a better accuracy and a clearer usefulness.

## Supplementary information

Supplementary material

## Data Availability

The dataset underlying this manuscript has been submitted to the European Genome-phenome Archive (EGA) under accession number EGAS00001005654.
