# Supplementary material for: A polygenic risk score for multiple myeloma risk prediction
Source: Eur J Hum Genet. 2021 Nov 30;30(4):474–9. doi: 10.1038/s41431-021-00986-8 (PMC8991223; doi:10.1038/s41431-021-00986-8)
Supplement: Supplementary file 1 — Supplementary material [file 41431_2021_986_MOESM1_ESM.docx]

**Supplementary table 1:** Selected SNPs.

| **SNP** | **Chr.** | **Position**  **(HG38)** | **Nearest gene** | **Data from Europeans in 1kG** | | **Data from GWAS papers** | | | | | **LD** |  |
| --- | --- | --- | --- | --- | --- | --- | --- | --- | --- | --- | --- | --- |
|  |  |  |  | **Major/minor**  **allele** | **MAF^a^** | **Reference/**  **risk allele** | **OR** | **P_value_** | **Reference** | **Original SNP** |  | |
| rs6746082 | 2 | 25,436,375 | *DTNB* | A/C | 0.21 | A/C | 1.29 | 1.22×10^-7^ | Broderick et al 2011 |  |  | |
| rs4325816 | 2 | 174,808,899 | *SP3* | T/C | 0.23 | T/C | 1.12 | 7.37×10^-9^ | Went et al 2018 |  |  | |
| rs1052501 | 3 | 41,883,906 | *ULK4* | T/C | 0.19 | C/T | 1.32 | 7.47×10^-9^ | Broderick et al 2011 |  |  | |
| rs10936599 | 3 | 169,774,313 | *MYNN* | G/A | 0.24 | G/A | 1.26 | 8.70×10^-14^ | Chubb et al 2013 |  |  | |
| rs2548594 | 5 | 95,908,710 | *ELL2* | C/T | 0.29 | C/T | 1.25 | 9.60×10^-10^ | Swaminathan et al 2015 | rs56219066 | 0.97 | |
| rs6595443 | 5 | 122,743,325 | *CEP120* | A/T | 0.45 | T/A | 1.11 | 1.20×10^-8^ | Went et al 2018 |  |  | |
| rs34229995 | 6 | 15,243,787 | *JARID2* | C/G | 0.02 | G/C | 1.37 | 1.31×10^-8^ | Mitchell et al 2016 |  |  | |
| rs2285803 | 6 | 31,139,481 | *PSORS1C1* | G/A | 0.26 | A/G | 1.19 | 9.67×10^-11^ | Chubb et al 2013 |  |  | |
| rs9373839 | 6 | 106,655,617 | *ATG5* | T/C | 0.19 | C/T | 1.18 | 9.19×10^-15^ | Mitchell et al 2016 | rs9372120 | 0.98 | |
| rs4487645 | 7 | 21,898,622 | *DNAH11* | C/A | 0.34 | C/A | 1.38 | 3.33×10^-15^ | Broderick et al 2011 |  |  | |
| rs17507636 | 7 | 106,291,118 | *CTB-30L5.1* | C/T | 0.26 | C/T | 1.12 | 9.20×10^-9^ | Went et al 2018 |  |  | |
| rs2170352 | 7 | 124,965,530 | *POT1-AS1* | G/A | 0.26 | G/A | 1.12 | 2.73×10^-8^ | Went et al 2018 | rs58618031 | 0.90 | |
| rs7781265 | 7 | 151,253,854 | *SMARCD3* | C/T | 0.09 | T/C | 1.19 | 9.71×10^-9^ | Mitchell et al 2016 |  |  | |
| rs1948915 | 8 | 127,210,176 | *CCAT1* | T/C | 0.33 | C/T | 1.13 | 4.20×10^-11^ | Mitchell et al 2016 |  |  | |
| rs2811710 | 9 | 21,991,924 | *CDKN2A* | G/A | 0.36 | G/A | 1.15 | 1.72×10^-13^ | Mitchell et al 2016 |  |  | |
| rs2790454 | 10 | 28,842,032 | *WAC* | A/G | 0.26 | A/G | 1.12 | 1.77×10^-8^ | Mitchell et al 2016 | rs2790457 | 1.00 | |
| rs7187359 | 16 | 30,691,834 |  | G/A | 0.28 | A/G | 1.15 | 1.02×10^-13^ | Went et al 2018 | rs13338946 | 0.96 | |
| rs7193541 | 16 | 74,630,845 | *RFWD3* | T/C | 0.39 | T/C | 1.13 | 5.00×10^-12^ | Mitchell et al 2016 |  |  | |
| rs4273077 | 17 | 16,945,825 | *TNFRSF13B* | A/G | 0.10 | G/A | 1.26 | 7.67×10^−9^ | Chubb et al 2013 |  |  | |
| rs11086029 | 19 | 16,438,661 | KLF2 | A/T | 0.23 | T/A | 1.14 | 6.79×10^-11^ | Went et al 2018 |  |  | |
| rs6066835 | 20 | 48,738,472 | *PREX1* | T/C | 0.09 | C/T | 1.26 | 1.36×10^-13^ | Mitchell et al 2016 |  |  | |
| rs138745 | 22 | 35,700,255 | *TOM1* | G/A | 0.34 | A/G | 1.18 | 5.70×10^-8^ | Swaminathan et al 2015 | rs138740 | 1.00 | |
| rs877529 | 22 | 39,146,287 | *CBX7* | G/A | 0.44 | A/G | 1.23 | 7.63×10^-16^ | Chubb et al 2013 |  |  | |

^a^ Data from European populations (EUR) in the 1000 Genome Project (http://www.ensembl.org/Homo_sapiens/Info/Index).

^b^ MAF: minor allele frequency; OR: odds ratio; CI: confidence interval.

^c^ SNPs for which a suitable genotyping assay was not available were replaced with surrogate SNP. This column reports the measure of linkage disequilibrium (LD) between each original SNP and its surrogate, expressed as r^2^ (data from European (EUR) populations in https://ldlink.nci.nih.gov).

**Supplementary table 2:** Example of score computation.

| **Subject** | **rs4487645** | **rs6746082** | **rs1052501** | **rs138745** | **rs877529** | **rs1948915** | **…** | **rs7193541** | **rs6595443** | **rs7781265** | **rs9373839** | **rs10936599** | **rs11086029** | **rs17507636** | **rs34229995** | **Call rate** | **Unweighted score** | **Percentile unw. score** | **Quintile unw. score** | **Unweighted score scaled** | **Percentile unw. score scaled** | **Quintile unw. Score scaled** | **Weighted score** | **Percentile wei. score** | **Quintile wei. score** | **Weighted score scaled** | **Percentile wei. score scaled** | **Quintile wei. score scaled** |
| --- | --- | --- | --- | --- | --- | --- | --- | --- | --- | --- | --- | --- | --- | --- | --- | --- | --- | --- | --- | --- | --- | --- | --- | --- | --- | --- | --- | --- |
| **Genotypes** |  |  |  |  |  |  |  |  |  |  |  |  |  |  |  |  |  |  |  |  |  |  |  |  |  |  |  |  |
| Control 1 | C/C | A/A | T/T | G/G | G/A | T/C | … | T/C | A/T | G/A | C/C | C/T | A/T | C/T | C/C | 100.0% |  |  |  |  |  |  |  |  |  |  |  |  |
| Control 2 | C/A | A/A | T/T | G/A | A/A | T/T | … | T/T | A/T | G/G | T/T | C/C | A/A | C/C | C/C | 100.0% |  |  |  |  |  |  |  |  |  |  |  |  |
| Control 3 | C/C | A/A | T/T | G/G | G/A | T/C | … | T/C | A/A | G/G | T/C | C/T | A/T | C/C | C/C | 100.0% |  |  |  |  |  |  |  |  |  |  |  |  |
| Control 4 | C/C | A/A | T/T | G/A | G/A | T/T | … | T/C | A/T | G/G | T/C | C/C | A/T | C/T | C/C | 100.0% |  |  |  |  |  |  |  |  |  |  |  |  |
| Control 5 | A/A | A/A | T/T | A/A | G/G | T/C | … | T/T | A/A | G/G | T/T | C/T | A/T | C/C | C/C | 100.0% |  |  |  |  |  |  |  |  |  |  |  |  |
| Control 6 | A/A | A/C | T/T | G/G | G/G | T/T | … | C/C | A/T | G/G | T/T | C/C | A/T | C/T | C/C | 95.7% |  |  |  |  |  |  |  |  |  |  |  |  |
| Control 7 | C/C | A/A | - | G/A | G/A | T/C | … | C/C | T/T | G/G | T/T | C/C | A/T | C/C | C/C | 95.7% |  |  |  |  |  |  |  |  |  |  |  |  |
| Control 8 | A/A | A/A | T/T | G/A | A/A | T/T | … | T/T | A/T | G/G | C/C | C/C | A/A | C/T | C/C | 100.0% |  |  |  |  |  |  |  |  |  |  |  |  |
| Control 9 | C/C | A/A | T/C | G/G | G/G | T/T | … | C/C | T/T | G/G | T/T | T/T | A/A | T/T | C/C | 95.7% |  |  |  |  |  |  |  |  |  |  |  |  |
| Control 10 | C/C | A/C | T/T | A/A | G/G | T/C | … | C/C | A/A | G/G | T/T | C/C | A/A | C/C | C/C | 100.0% |  |  |  |  |  |  |  |  |  |  |  |  |
| Case 1 | A/A | A/A | T/T | G/G | G/A | T/T | … | T/T | A/T | G/A | T/T | C/T | A/A | C/T | C/C | 100.0% |  |  |  |  |  |  |  |  |  |  |  |  |
| Case 2 | C/C | A/A | T/T | G/A | G/G | T/T | … | T/C | A/T | G/G | T/C | C/T | A/T | T/T | C/C | 100.0% |  |  |  |  |  |  |  |  |  |  |  |  |
| Case 3 | C/C | A/C | T/C | A/A | A/A | T/C | … | C/C | A/A | G/G | T/C | C/T | A/A | C/T | C/C | 100.0% |  |  |  |  |  |  |  |  |  |  |  |  |
| Case 4 | A/A | A/C | T/T | G/A | G/G | T/T | … | T/T | T/T | G/G | T/C | C/T | A/A | C/C | C/C | 100.0% |  |  |  |  |  |  |  |  |  |  |  |  |
| Case 5 | C/C | A/C | T/T | G/G | G/A | T/T | … | T/T | A/T | G/G | T/T | T/T | T/T | C/C | C/C | 100.0% |  |  |  |  |  |  |  |  |  |  |  |  |
| Case 6 | C/A | A/A | T/T | G/G | G/G | T/C | … | T/T | A/A | G/A | T/T | C/C | A/A | C/T | C/C | 100.0% |  |  |  |  |  |  |  |  |  |  |  |  |
| Case 7 | C/C | A/A | T/T | A/A | G/G | C/C | … | T/T | A/A | G/G | T/T | C/C | - | C/T | C/C | 95.7% |  |  |  |  |  |  |  |  |  |  |  |  |
| Case 8 | A/A | A/C | T/C | G/A | G/A | T/T | … | C/C | A/T | G/G | T/C | C/C | A/A | C/T | C/C | 100.0% |  |  |  |  |  |  |  |  |  |  |  |  |
| Case 9 | C/A | A/C | T/C | G/G | G/G | T/C | … | T/T | T/T | G/G | T/C | C/T | A/T | C/C | C/C | 100.0% |  |  |  |  |  |  |  |  |  |  |  |  |
| Case 10 | C/C | A/A | T/T | G/G | G/A | T/T | … | T/C | A/A | A/A | T/C | C/T | A/T | C/T | C/C | 100.0% |  |  |  |  |  |  |  |  |  |  |  |  |
| **Number of risk alleles** | | |  |  |  |  |  |  |  |  |  |  |  |  |  |  |  |  |  |  |  |  |  |  |  |  |  |  |
| Control 1 | 2 | 2 | 0 | 0 | 1 | 1 |  | 1 | 1 | 1 | 2 | 1 | 1 | 1 | 0 | 100.0% | 24 | 0.95 | 5 | 24.00 | 0.89 | 5 |  |  |  |  |  |  |
| Control 2 | 1 | 2 | 0 | 1 | 2 | 0 |  | 2 | 1 | 0 | 0 | 2 | 0 | 2 | 0 | 100.0% | 20 | 0.42 | 3 | 20.00 | 0.42 | 3 |  |  |  |  |  |  |
| Control 3 | 2 | 2 | 0 | 0 | 1 | 1 |  | 1 | 0 | 0 | 1 | 1 | 1 | 2 | 0 | 100.0% | 19 | 0.26 | 2 | 19.00 | 0.26 | 2 |  |  |  |  |  |  |
| Control 4 | 2 | 2 | 0 | 1 | 1 | 0 |  | 1 | 1 | 0 | 1 | 2 | 1 | 1 | 0 | 100.0% | 21 | 0.58 | 3 | 21.00 | 0.58 | 3 |  |  |  |  |  |  |
| Control 5 | 0 | 2 | 0 | 2 | 0 | 1 |  | 2 | 0 | 0 | 0 | 1 | 1 | 2 | 0 | 100.0% | 20 | 0.42 | 3 | 20.00 | 0.42 | 3 |  |  |  |  |  |  |
| Control 6 | 0 | 1 | 0 | 0 | 0 | 0 |  | 0 | 1 | 0 | 0 | 2 | 1 | 1 | 0 | 95.7% | 15 | 0.05 | 1 | 15.68 | 0.11 | 1 |  |  |  |  |  |  |
| Control 7 | 2 | 2 |  | 1 | 1 | 1 |  | 0 | 2 | 0 | 0 | 2 | 1 | 2 | 0 | 95.7% | 23 | 0.84 | 5 | 24.05 | 1.00 | 5 |  |  |  |  |  |  |
| Control 8 | 0 | 2 | 0 | 1 | 2 | 0 |  | 2 | 1 | 0 | 2 | 2 | 0 | 1 | 0 | 100.0% | 22 | 0.68 | 4 | 22.00 | 0.68 | 4 |  |  |  |  |  |  |
| Control 9 | 2 | 2 | 1 | 0 | 0 | 0 |  | 0 | 2 | 0 | 0 | 0 | 0 | 0 | 0 | 95.7% | 14 | 0.00 | 1 | 14.64 | 0.00 | 1 |  |  |  |  |  |  |
| Control 10 | 2 | 1 | 0 | 2 | 0 | 1 |  | 0 | 0 | 0 | 0 | 2 | 0 | 2 | 0 | 100.0% | 19 | 0.26 | 2 | 19.00 | 0.26 | 2 |  |  |  |  |  |  |
| Case 1 | 0 | 2 | 0 | 0 | 1 | 0 |  | 2 | 1 | 1 | 0 | 1 | 0 | 1 | 0 | 100.0% | 17 | 0.16 | 1 | 17.00 | 0.16 | 1 |  |  |  |  |  |  |
| Case 2 | 2 | 2 | 0 | 1 | 0 | 0 |  | 1 | 1 | 0 | 1 | 1 | 1 | 0 | 0 | 100.0% | 21 | 0.58 | 3 | 21.00 | 0.58 | 3 |  |  |  |  |  |  |
| Case 3 | 2 | 1 | 1 | 2 | 2 | 1 |  | 0 | 0 | 0 | 1 | 1 | 0 | 1 | 0 | 100.0% | 24 | 0.95 | 5 | 24.00 | 0.89 | 5 |  |  |  |  |  |  |
| Case 4 | 0 | 1 | 0 | 1 | 0 | 0 |  | 2 | 2 | 0 | 1 | 1 | 0 | 2 | 0 | 100.0% | 20 | 0.42 | 3 | 20.00 | 0.42 | 3 |  |  |  |  |  |  |
| Case 5 | 2 | 1 | 0 | 0 | 1 | 0 |  | 2 | 1 | 0 | 0 | 0 | 2 | 2 | 0 | 100.0% | 23 | 0.84 | 5 | 23.00 | 0.79 | 4 |  |  |  |  |  |  |
| Case 6 | 1 | 2 | 0 | 0 | 0 | 1 |  | 2 | 0 | 1 | 0 | 2 | 0 | 1 | 0 | 100.0% | 18 | 0.21 | 2 | 18.00 | 0.21 | 2 |  |  |  |  |  |  |
| Case 7 | 2 | 2 | 0 | 2 | 0 | 2 |  | 2 | 0 | 0 | 0 | 2 |  | 1 | 0 | 95.7% | 22 | 0.68 | 4 | 23.00 | 0.79 | 4 |  |  |  |  |  |  |
| Case 8 | 0 | 1 | 1 | 1 | 1 | 0 |  | 0 | 1 | 0 | 1 | 2 | 0 | 1 | 0 | 100.0% | 15 | 0.05 | 1 | 15.00 | 0.05 | 1 |  |  |  |  |  |  |
| Case 9 | 1 | 1 | 1 | 0 | 0 | 1 |  | 2 | 2 | 0 | 1 | 1 | 1 | 2 | 0 | 100.0% | 22 | 0.68 | 4 | 22.00 | 0.68 | 4 |  |  |  |  |  |  |
| Case 10 | 2 | 2 | 0 | 0 | 1 | 0 |  | 1 | 0 | 2 | 1 | 1 | 1 | 1 | 0 | 100.0% | 19 | 0.26 | 2 | 19.00 | 0.26 | 2 |  |  |  |  |  |  |
| Weights |  |  |  |  |  |  |  |  |  |  |  |  |  |  |  |  |  |  |  |  |  |  |  |  |  |  |  |  |
| Ln(OR_het_^a^) | 0.30 | 0.10 | 0.36 | 0.08 | 0.19 | 0.12 |  | 0.14 | 0.25 | 0.05 | 0.09 | 0.18 | 0.14 | 0.24 | 0.22 | 100.0% |  |  |  |  |  |  |  |  |  |  |  |  |
| Ln(OR_hom_^a^) | 0.67 | 0.28 | 0.41 | 0.21 | 0.41 | 0.32 |  | 0.13 | 0.25 | 0.30 | 0.10 | 0.36 | 0.27 | 0.53 | 1.00 | 100.0% |  |  |  |  |  |  |  |  |  |  |  |  |
| Control 1 | 0.67 | 0.28 | 0.00 | 0.00 | 0.19 | 0.12 |  | 0.14 | 0.25 | 0.05 | 0.10 | 0.18 | 0.14 | 0.24 | 0.00 | 100.0% |  |  |  |  |  |  | 3.80 | 1.00 | 5 | 3.80 | 1.00 | 5 |
| Control 2 | 0.30 | 0.28 | 0.00 | 0.08 | 0.41 | 0.00 |  | 0.13 | 0.25 | 0.00 | 0.00 | 0.36 | 0.00 | 0.53 | 0.00 | 100.0% |  |  |  |  |  |  | 3.16 | 0.63 | 4 | 3.16 | 0.63 | 4 |
| Control 3 | 0.67 | 0.28 | 0.00 | 0.00 | 0.19 | 0.12 |  | 0.14 | 0.00 | 0.00 | 0.09 | 0.18 | 0.14 | 0.53 | 0.00 | 100.0% |  |  |  |  |  |  | 3.05 | 0.53 | 3 | 3.05 | 0.53 | 3 |
| Control 4 | 0.67 | 0.28 | 0.00 | 0.08 | 0.19 | 0.00 |  | 0.14 | 0.25 | 0.00 | 0.09 | 0.36 | 0.14 | 0.24 | 0.00 | 95.7% |  |  |  |  |  |  | 3.54 | 0.79 | 4 | 3.69 | 0.85 | 4 |
| Control 5 | 0.00 | 0.28 | 0.00 | 0.21 | 0.00 | 0.12 |  | 0.13 | 0.00 | 0.00 | 0.00 | 0.18 | 0.14 | 0.53 | 0.00 | 95.7% |  |  |  |  |  |  | 2.55 | 0.26 | 2 | 2.66 | 0.30 | 2 |
| Control 6 | 0.00 | 0.10 | 0.00 | 0.00 | 0.00 | 0.00 |  | 0.00 | 0.25 | 0.00 | 0.00 | 0.36 | 0.14 | 0.24 | 0.00 | 100.0% |  |  |  |  |  |  | 2.11 | 0.05 | 1 | 2.16 | 0.05 | 1 |
| Control 7 | 0.67 | 0.28 | 0.00 | 0.08 | 0.19 | 0.12 |  | 0.00 | 0.25 | 0.00 | 0.00 | 0.36 | 0.14 | 0.53 | 0.00 | 95.7% |  |  |  |  |  |  | 3.74 | 0.89 | 5 | 3.96 | 0.95 | 5 |
| Control 8 | 0.00 | 0.28 | 0.00 | 0.08 | 0.41 | 0.00 |  | 0.13 | 0.25 | 0.00 | 0.10 | 0.36 | 0.00 | 0.24 | 0.00 | 100.0% |  |  |  |  |  |  | 2.68 | 0.32 | 2 | 2.68 | 0.32 | 2 |
| Control 9 | 0.67 | 0.28 | 0.36 | 0.00 | 0.00 | 0.00 |  | 0.00 | 0.25 | 0.00 | 0.00 | 0.00 | 0.00 | 0.00 | 0.00 | 100.0% |  |  |  |  |  |  | 1.99 | 0.00 | 1 | 2.04 | 0.00 | 1 |
| Control 10 | 0.67 | 0.10 | 0.00 | 0.21 | 0.00 | 0.12 |  | 0.00 | 0.00 | 0.00 | 0.00 | 0.36 | 0.00 | 0.53 | 0.00 | 100.0% |  |  |  |  |  |  | 2.91 | 0.47 | 3 | 2.91 | 0.42 | 3 |
| Case 1 | 0.00 | 0.28 | 0.00 | 0.00 | 0.19 | 0.00 |  | 0.13 | 0.25 | 0.05 | 0.00 | 0.18 | 0.00 | 0.24 | 0.00 | 100.0% |  |  |  |  |  |  | 2.43 | 0.16 | 1 | 2.43 | 0.16 | 1 |
| Case 2 | 0.67 | 0.28 | 0.00 | 0.08 | 0.00 | 0.00 |  | 0.14 | 0.25 | 0.00 | 0.09 | 0.18 | 0.14 | 0.00 | 0.00 | 100.0% |  |  |  |  |  |  | 3.27 | 0.74 | 4 | 3.27 | 0.74 | 4 |
| Case 3 | 0.67 | 0.10 | 0.36 | 0.21 | 0.41 | 0.12 |  | 0.00 | 0.00 | 0.00 | 0.09 | 0.18 | 0.00 | 0.24 | 0.00 | 100.0% |  |  |  |  |  |  | 3.77 | 0.95 | 5 | 3.77 | 0.89 | 5 |
| Case 4 | 0.00 | 0.10 | 0.00 | 0.08 | 0.00 | 0.00 |  | 0.13 | 0.25 | 0.00 | 0.09 | 0.18 | 0.00 | 0.53 | 0.00 | 100.0% |  |  |  |  |  |  | 2.73 | 0.37 | 2 | 2.73 | 0.37 | 2 |
| Case 5 | 1.96 | 1.11 | 1.00 | 1.00 | 1.21 | 1.00 |  | 1.14 | 1.29 | 1.00 | 1.00 | 1.00 | 1.31 | 1.70 | 1.00 | 95.7% |  |  |  |  |  |  | 3.70 | 0.84 | 5 | 3.87 | 1.00 | 5 |
| Case 6 | 1.35 | 1.32 | 1.00 | 1.00 | 1.00 | 1.13 |  | 1.14 | 1.00 | 1.05 | 1.00 | 1.43 | 1.00 | 1.27 | 1.00 | 100.0% |  |  |  |  |  |  | 2.34 | 0.11 | 1 | 2.34 | 0.11 | 1 |
| Case 7 | 1.96 | 1.32 | 1.00 | 1.23 | 1.00 | 1.38 |  | 1.14 | 1.00 | 1.00 | 1.00 | 1.43 | 1.00 | 1.27 | 1.00 | 100.0% |  |  |  |  |  |  | 2.87 | 0.42 | 3 | 2.92 | 0.47 | 3 |
| Case 8 | 1.00 | 1.11 | 1.43 | 1.08 | 1.21 | 1.00 |  | 1.00 | 1.29 | 1.00 | 1.09 | 1.43 | 1.00 | 1.27 | 1.00 | 100.0% |  |  |  |  |  |  | 2.43 | 0.21 | 2 | 2.43 | 0.21 | 2 |
| Case 9 | 1.35 | 1.11 | 1.43 | 1.00 | 1.00 | 1.13 |  | 1.14 | 1.29 | 1.00 | 1.09 | 1.20 | 1.15 | 1.70 | 1.00 | 100.0% |  |  |  |  |  |  | 3.23 | 0.68 | 4 | 3.23 | 0.68 | 4 |
| Case 10 | 1.96 | 1.32 | 1.00 | 1.00 | 1.21 | 1.00 |  | 1.15 | 1.00 | 1.35 | 1.09 | 1.20 | 1.15 | 1.27 | 1.00 | 100.0% |  |  |  |  |  |  | 3.07 | 0.58 | 3 | 3.07 | 0.58 | 3 |

^a^ OR_het_: odds ratio in heterozygotes, compared with homozygotes for the non-risk allele; OR_hom_: odds ratio in homozygotes for the risk allele, compared with homozygotes for the non-risk allele. These ORs were computed in the IMMEnSE population, and used as weights for the setup of the weighted score.

**Supplementary figure. 1**. Risk allele distribution between MM cases and controls in IMMEnSE *.


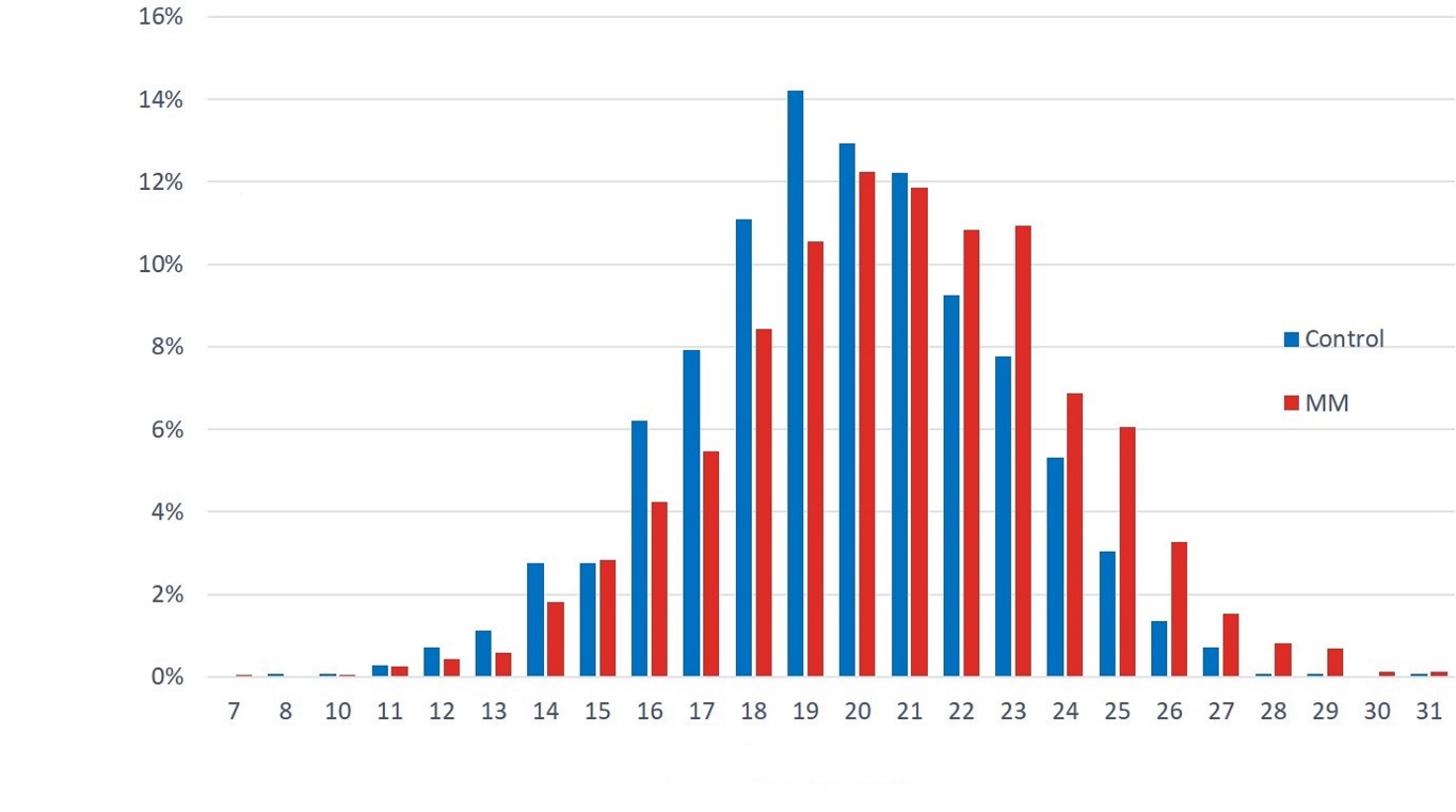


*X axis=N° of risk alleles; Y axis=frequency in the population.
